# Forced intracellular degradation of xenoantigens as a modality for cell-based cancer immunotherapy

**DOI:** 10.1016/j.isci.2025.111957

**Published:** 2025-02-04

**Authors:** Jean Pierre Bikorimana, Roudy Farah, Jamilah Abusarah, Gabrielle Arona Mandl, Mohamed Ali Erregragui, Marina Pereira Gonçalves, Sebastien Talbot, Perla Matar, Malak Lahrichi, Nehme El-Hachem, Moutih Rafei

**Affiliations:** 1 Department of Microbiology, Infectious Diseases and Immunology, Université de Montréal, Montréal, QC H3T1J4, Canada; 2 Department of Pharmacology and Physiology, Université de Montréal, Montréal, QC H3T1J4, Canada; 3 Molecular Biology Program, Université de Montréal, Montréal, QC H3T1J4, Canada; 4 Department of Biomedical and Molecular Sciences, Queen’s University, Kingston, ON K7L3N6, Canada; 5 Pediatric Hematology-Oncology Division, Centre Hospitalier Universitaire Sainte-Justine Research Centre, Montreal, QC H3T1C5, Canada

**Keywords:** Cellular therapy, Immunology, Cancer

## Abstract

Given recent leverage of mesenchymal stromal cells (MSCs) as a potent vaccination platform, we investigated whether forced degradation of an expressed experimental antigen fused to small degron sequences could prime potent antitumoral responses. Retrovirally gene-engineered MSCs were evaluated for their *in-vitro* antigen presentation capacity, nature of generated peptide repertoire and therapeutic potency in syngeneic immunocompetent mice with pre-established solid T cell lymphoma. Despite lack of noticeable changes in gene expression, MSC-UBvR-OVA vaccination triggered potent T cell activation which can be attributable to the enriched cell surface presentation of OVA-derived peptides added to elevated mitochondrial reactive oxidative species (ROS) production, the latter being associated with efficient antigen processing. Where MSC-UBvR-OVA vaccination successfully controlled tumor growth in cancer-bearing mice, the effect is further enhanced using tranylcypromine-stimulated MSCs and anti-PD-1 combination. Such anti-tumoral response relies on efferocytosis by endogenous phagocytes. Altogether, UBvR facilitated forced antigen degradation represents a plausible modality for future development of tumor antigen-expressing MSC-based vaccine.

## Highlights

• The UBvR degron is superior to RNP4 1–80 is promoting OVA degradation
• The antigen presenting ability of MSC-UBvR-OVA cells requires ROS production
• The MSC-UBvR-OVA cells synergize with anti-PD-1 in priming antitumoral activity

## Graphical abstract

**Figure:**
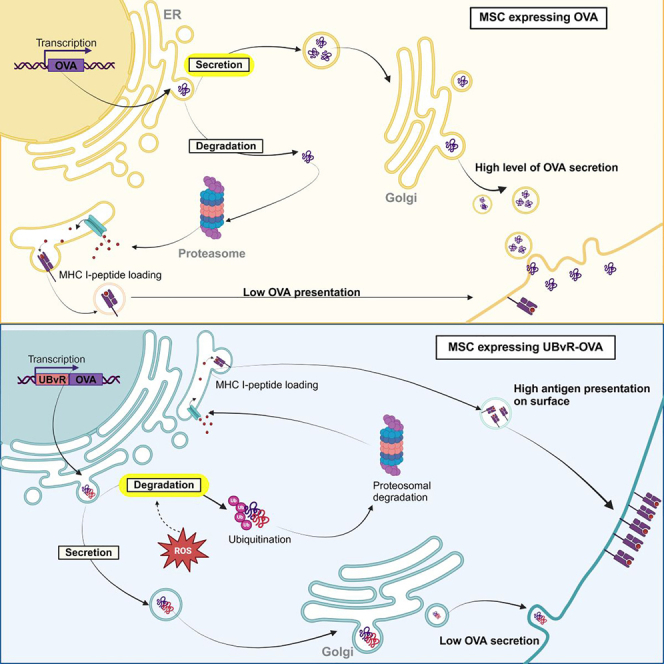

## Introduction

The development of anticancer vaccines and immune checkpoint inhibitors (ICI) to boost antitumoral immunity marks a hallmark advancement revolutionizing the clinical management of cancer.^1^ Nonetheless, limited clinical effectiveness of the currently available Food and Drug Administration (FDA)-approved cancer vaccine, Provenge,^2^ highlights the need to revisit and optimize the main components of a successful anticancer vaccine: antigen processing and presentation.^3^^,^^4^ These steps require target antigens, whether produced endogenously or engulfed by an antigen-presenting cell (APC), to be processed into 8–11 amino acid peptides suitable for generating stable major histocompatibility complex (MHC) I-antigen complex for CD8^+^ T cell priming.^5^^,^^6^ Within APCs, captured antigens get sorted into endosomes where they undergo partial degradation by proteases before their release/leaking into in the cytosol where they undergo further processing by the proteasomal complex to generate antigenic peptides.^7^^,^^8^ However, a significant portion of the antigen is lost during endosomal maturation due to excessive and/or unwanted degradation by extreme acidity and resident activated proteases.^9^^,^^10^^,^^11^^,^^12^^,^^13^^,^^14^ On the other hand, endogenously produced target proteins exiting the endoplasmic-reticulum (ER) traffic via the Golgi apparatus for exocytosis resulting in their release into the extracellular space.^15^ Loss of antigen via either case limits its intracellular amount available for processing and loading onto MHC I molecules. To address this pitfall, targeted delivery of the antigen toward the proteasome machinery may be achieved using specific degron sequences. Degrons have been extensively studied as tools for manipulating protein levels or for regulating protein degradation rates.^16^^,^^17^^,^^18^ Interestingly, degrons include well preserved amino acid sequences varying in size from single amino acids, to short peptides, post-translational structural motifs, or exposed amino acids.^19^ Depending on the present degron, the tagged or modified protein may be directed for degradation via two main pathways: (1) the ubiquitin-proteasome pathway where polyubiquitinated proteins are identified by either of the proteasome regulatory particles (Rpn) 10, Rpn13, and Rpn1 and directed to the 26S proteasome for degradation,^20^^,^^21^^,^^22^ or (2) the ubiquitin-independent proteolysis pathway.^23^ Degrons belonging to the latter pathway such as the first 80 residues of transcription factor Rnp4 N-terminal region may be recognized by specific 19S RP subunits, but their detailed mode of action remains poorly understood.^23^

To translate the benefits of improved antigen targeting into effective immune response, the second component focuses on the utilized APC. Unfortunately, the use of *ex vivo* generated monocyte-derived dendritic (DCs) cells for cellular vaccination showed limited effectiveness in the clinic due to impaired migration and antigen presentation capacity as well as weak persistence *in vivo.*^24^^,^^25^^,^^26^ We have previously introduced and extensively studied gene-engineered or pharmacologically reprogrammed MSCs as a cell-based platform suitable for cancer vaccine development.^27^^,^^28^^,^^29^^,^^30^^,^^31^^,^^32^ In line with the current research directions in cancer therapy,^33^ it has become increasingly evident that immunotherapies would benefit from employing multiple technologies for precise antigen targeting to the cell of interest.^34^^,^^35^^,^^36^^,^^37^ For example, nano-encapsulation, aided by the specific targeting abilities of monoclonal antibodies are wildly studied approaches to improve the delivery of chemotherapeutic agents, peptides and gene editing tools to cancer cells, APCs or genetically defected cell respectively.^38^^,^^39^^,^^40^^,^^41^^,^^42^ In cell-based cancer vaccination, employing multiplex effective *in vivo* targeting strategies to improve antigen delivery and internalization coupled with effective intracellular antigen processing can significantly improve vaccine outcome, thus bypassing the necessity for *ex vivo* DC culturing.^43^^,^^44^^,^^45^ We thus evaluated the outcome of engineering MSCs to express destabilized forms of the ovalbumin (OVA) protein using two different degrons: UBvR and Rnp4 1–80. This strategy could allow a direct comparison for MSCs antigen presentation properties when the protein is targeted to the proteasome via ubiquitin-dependent versus independent pathways respectively.

## Results

### The UBvR degron elicits stronger T cell activation

**Figure 1:**
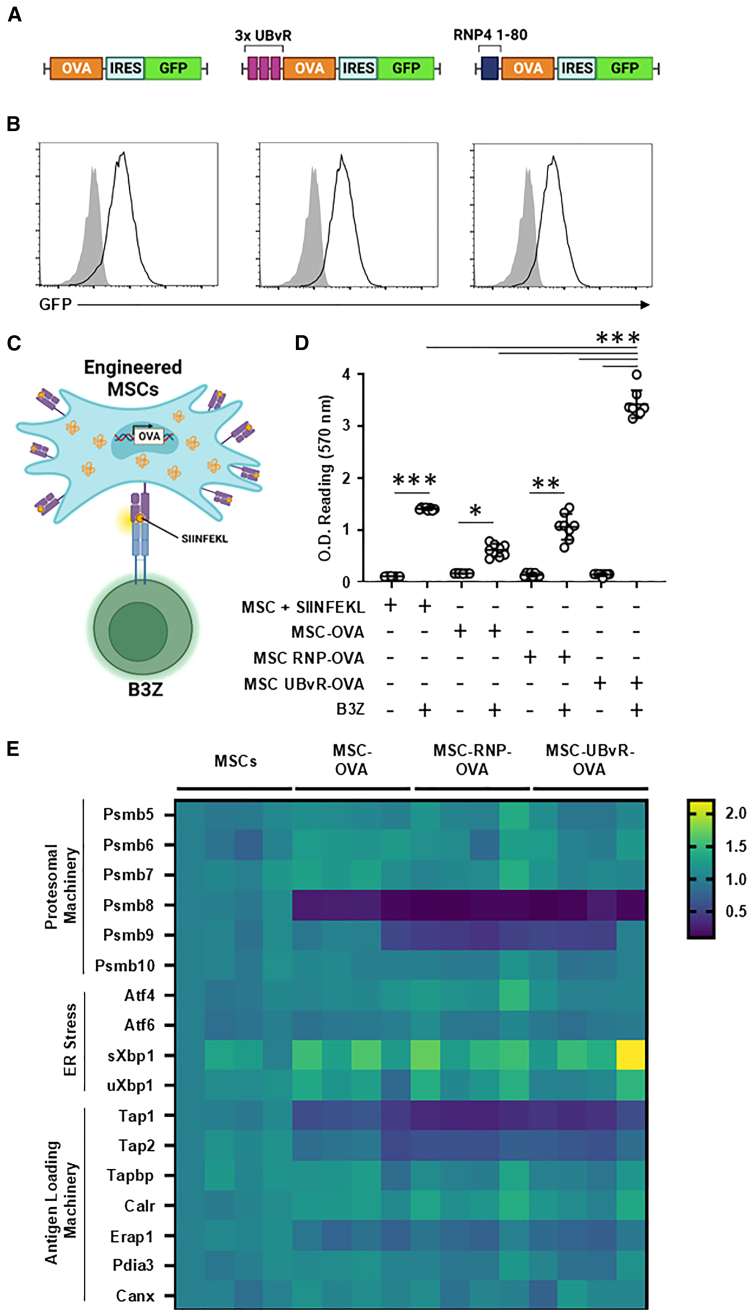
Characterizing the antigen presenting properties of gene-engineered MSCs (A) Cartoons of the three OVA cDNA constructs. (B) Representative flow cytometry assessment of eGFP expression following transduction using the three constructs listed in (A). (C) A cartoon reflecting the antigen presentation assay used in the study. (D) Antigen presentation assay using the B3Z T cell line co-cultured with MSC-OVA, MSC-UBvR-OVA or MSC-RNP4 1-80-OVA. SIINFEKL pulsed MSCs were used as positive controls. (E) A heatmap depicting the expression profile of several genes related to proteasomal degradation, ER stress and antigen presentation. The heatmap was generated using the GraphPad Prism 10 software (version 10.2.1 – www.graphpad.com). For (D) and (E), *n* = 6 and *n* = 4/group, respectively with ∗∗∗*p* < 0.001. See also Figures S1–S3. Data are represented as mean ± SD.

Expression of a given antigen in a cell can lead to two outcomes: intracellular degradation via the proteasome and/or extracellular secretion of the protein. To override the secretion pathway, MSCs were gene-engineered to express OVA cDNAs containing two different degrons sequences. While the first degron sequence (3 repeats of the UBvR sequence) relies on ubiquitination, the second one (3 repeats of the RNP4 1–80 sequence) functions in an ubiquitin-independent manner through its recruitment via 19S RP subunits (Rpt1, Rpn2, and Rpn5—Figures 1A and S1).^23^^,^^46^ Following confirmation of efficient transduction, equal transgene expression through eGFP assessment by flow cytometry (Figure 1B) and retained MSC phenotype (Figure S2), an antigen presentation assay was conducted to compare the impact that the degron sequences may have on SIINFEKL (OVA-derived peptide) presentation by the H2-K^b^ MHC I molecule (Figure 1C). Compared to MSC-OVA (SIINFEKL-pulsed MSCs were used as a positive control for the assay), the MSC-UBvR-OVA group triggered the strongest B3Z (CD8 T cell hybridoma recognizing the SIINFEKL peptide presented by H2-K^b^) response followed by MSC-RNP-OVA (Figure 1D). Similar results were obtained in antigen presentation assay using the standard OT-I-derived primary CD8^+^ T cells (Figure S3). In an attempt to understand the reason behind such improved T cell activation, quantification of several gene transcripts involved in the proteasomal machinery, ER stress, or antigen loading was conducted. Besides trivial decreased expression of some immunoproteasome subunits (*Psmb8* and *Psmb9*), and two common antigen loading proteins (*Tap1* and *Tap2*), only one unfolded protein response gene related to ER stress (short *Xbp1*) was shown to be enhanced in all gene-engineered MSC groups (Figure 1E). The fact that improved *in vitro* B3Z T cell response by MSC-UBvR-OVA could not be explained by distinct gene expression profiles suggests that the UBvR degron may potentially enhance antigen presentation through a process independent of the endogenous machinery related to proteasome-based processing and loading on MHC I molecules. Interestingly enough, the latter process was reported to occur in transporter associated with antigen processing (TAP)-deficient DCs via an unknown peptide transporter capable of replacing TAP-dependent MHC I loading.^47^

### The UBvR degron enriches OVA-derived peptide presentation

**Figure 2:**
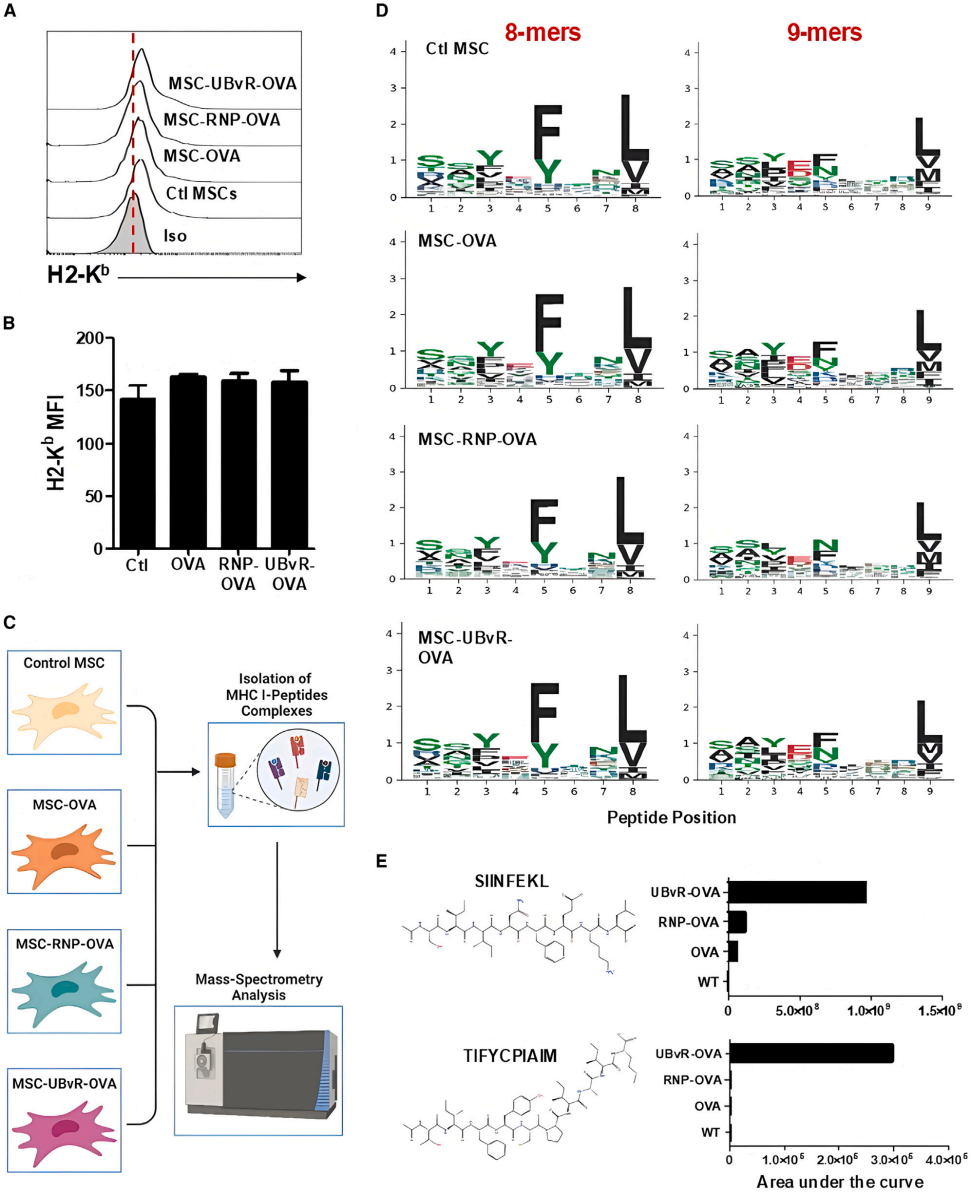
MSC-UBvR-OVA presents enriched OVA-derived peptides via MHCI (A) Flow cytometry analysis of H2-K^b^ on the different MSC populations. (B) The mean fluorescent intensity (MFI) of the H2-K^b^ analysis shown in (A). (C) A cartoon representing the study design for the immunopeptidome study using mass spectrometry. (D) Sequence logos showing the peptide-binding motifs of H2-K^b^ for the 8- and 9-mers peptides on the different MSC populations that were identified using mass spectrometry. (E) Analysis of the two OVA-derived peptides presented on the different MSC populations based on the analysis conducted using the immunopeptidome study. For (A)–(D), *n* = 4/group. Data are represented as mean ± SD.

Given the lack of distinct gene expression in the MSC-UBvR-OVA population, we next wondered whether the elevated T cell response induced by MSC-UBvR-OVA was due to enhanced cell surface expression of H2-K^b^. Although the overall H2-K^b^ cell surface level was very low, we found no major differences among all studied populations (Figures 2A and 2B). This led us to speculate that the enhanced response observed earlier could potentially be caused by a difference in the peptide repertoire quality. To test this hypothesis, an immunopeptidome study was designed where H2-K^b^-peptide complexes isolated from each of the tested MSC groups were analyzed by mass spectrometry to identify and quantify OVA-derived peptides (Figure 2C). Analysis of the peptide motifs for H2-K^b^ revealed shared common hydrophobic amino acid at the 5^th^ and C-terminal anchor positions for both 8- and 9-mers long peptides (Figure 2D). Interestingly however, mining of the OVA-derived peptides on collected H2-K^b^-peptide complexes revealed an enrichment for the SIINFEKL (by at least 20-folds compared to the remaining groups) and another OVA-derived peptide (TIFYCPIAIM), which was only detected on MSC-UBvR-OVA (Figure 2E). These results indicate that despite the absence of modulated gene expression or increased cell surface H2-K^b^, the SIINFEKL peptide is enriched on the surface of the MSC population containing the UBvR degron sequence.

### Protein destabilization using the UBvR degron affects key biological processes related to protein modification and Tp53 activity

**Figure 3:**
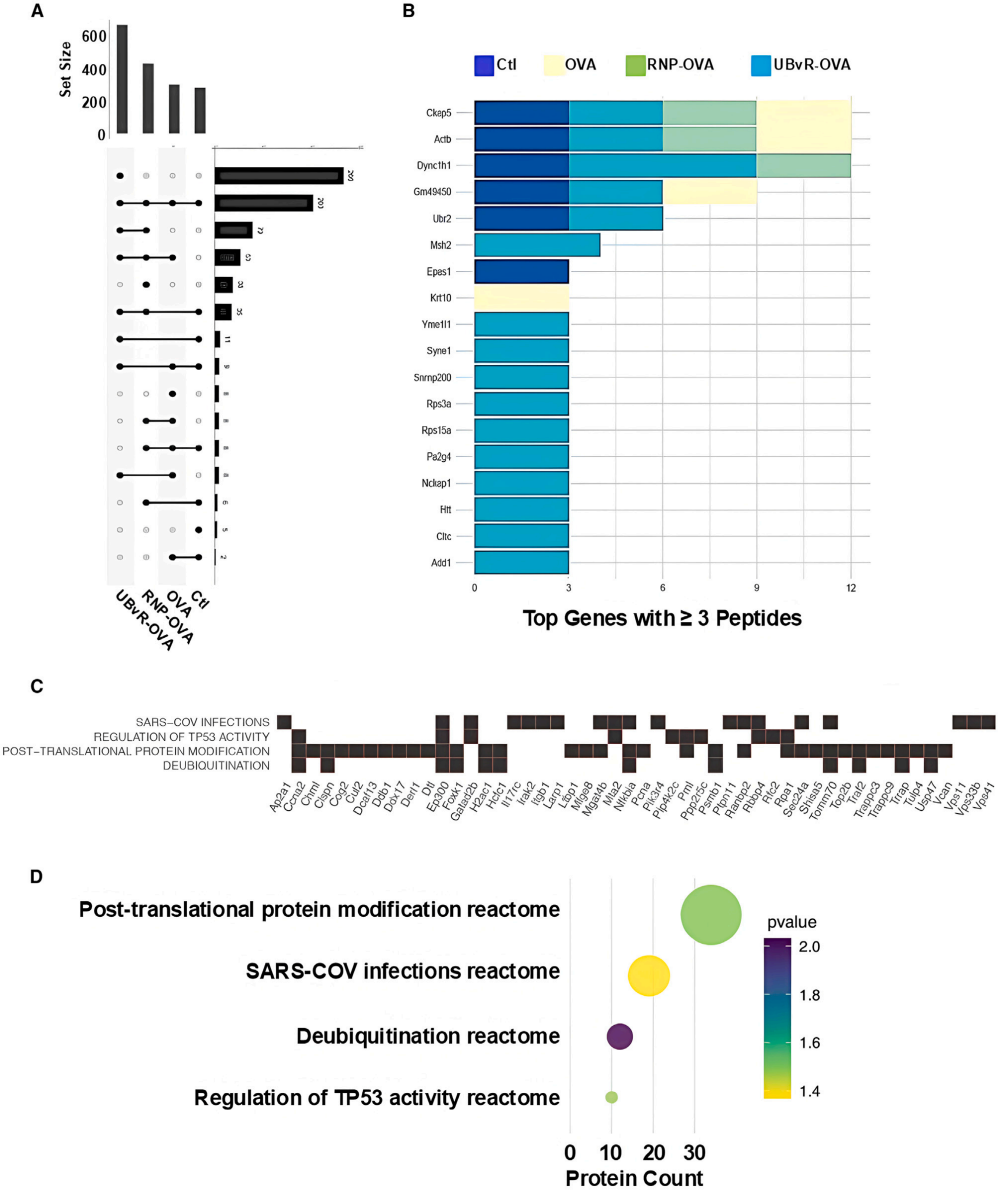
Data-driven analyses of the immunopeptidome in the MSC populations (A) To visualize the interplay of unique peptides in the immunopeptidome experiment, the upset plot illustrates the overlap of unique peptides between all four groups: MSC-UbvR-OVA, MSC-RNP-OVA, MSC-OVA, and control MSCs. (B) The bar plot illustrates the distribution of peptides associated with mouse genes, with a minimum threshold of three or more peptides. Each bar on the plot represents a unique mouse gene, identified on the y axis, while the x axis displays the count of peptides. (C) The heatmap illustrates the enrichment of reactome pathways in unique genes/peptides specific to the MSC-UBvR-OVA group, as compared to all other experimental contrasts. The genes responsible for this statistical enrichment are displayed along the x axis. (D) A dot plot representing the *p* values of the four significant processes shown in (C) alongside the count of proteins/peptides. Data are represented as mean ± SD.

Computational analysis of the immunopeptidome across the four MSC groups revealed several key insights. First, we identified a conserved set of 203 peptides shared among all four models (Figure 3A). Additionally, 266 peptides were uniquely detected in MSC-UBvR-OVA, 38 in MSC-RNP-OVA, 8 in MSC-OVA, and 5 in control cells (Figure 3A). To further investigate the origin of these peptides, we focused on proteins contributing at least three peptides across all groups (Figure 3B), leading to the identification of 18 proteins. Notably, 10 of these 18 proteins were exclusively detected in MSC-UBvR-OVA, while the remaining 8 were increasingly represented in control, MSC-RNP-OVA, and MSC-OVA groups, respectively. A detailed analysis of the 266 peptides unique to MSC-UBvR-OVA identified four significantly enriched pathways (*p* < 0.05), as determined by pathway enrichment analysis (Figure 3C). Among these pathways, the SARS-CoV infection pathway likely reflects a general overlap in protein responses with the UBvR group. The other three pathways were associated with critical biological processes, including Tp53 activity regulation, deubiquitination, and protein post-translational modification. These findings suggest that the UBvR degron induces the largest repertoire of unique peptides, followed by RNP4, as visualized in a reactome pathway overrepresentation analysis (Figure 3D). The four UBvR-related pathways showed statistical significance relative to the background peptide/protein set, with the contributing protein counts highlighted in Figure 3D. In addition to modulating ubiquitination and post-translational modifications, as expected from the degron insertion, these data indicate that protein destabilization mediated by the UBvR degron may also influence Tp53 activity, potentially through the induction of genotoxic stress.

### The antigen presentation capability of MSC-UBvR-OVA relies on mitochondrial-derivedROS

**Figure 4:**
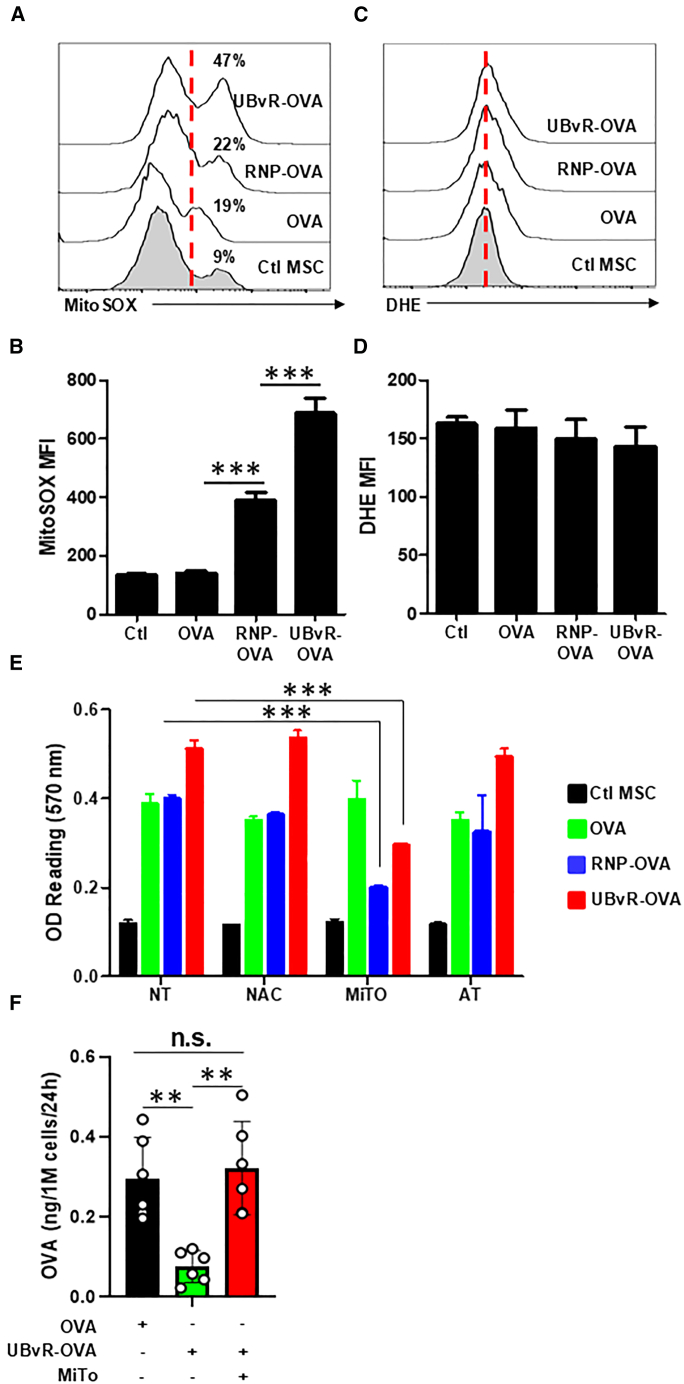
UBvR-mediated antigen presentation relies on mitochondrial-derived ROS (A) Flow cytometry analysis of mitochondrial-derived ROS using MitoSOX staining. (B) MFI analysis of the signal obtained in (A). (C) Flow cytometry analysis of cytoplasmic ROS using DHE staining. (D) MFI analysis of the signal obtained in (C). (E) Antigen presentation assay using the different MSC populations following their treatment with anti-oxidants. The color codes are as follow: Ctl MSCs (black); MSC-OVA (green); MSC-RNP-OVA (blue); and MSC-UBvR-OVA (red). The different treatment groups are non-treated cells (NT), treatment with N-acetylcysteine (NAC), treatment with MitoTEMPO (MiTO); treatment with α-tocopherol (AT). (F) ELISA-based quantification of the OVA protein in the supernatant of MSC-OVA or MSC-UBvR-OVA in the presence or absence of MitoTEMPO. For this panel, *n* = 5/group with ∗∗∗*p* < 0.001. Data are represented as mean ± SD.

Although reactive oxidative species (ROS) production is always perceived as a negative byproduct of oxidative phosphorylation, ROS are involved in antigen cross-/presentation.^48^ For instance, ROS could block endosomal acidification after antigen capture decreasing non-specific damages inflicted by the activation of intra-endosomal proteases.^12^ In addition, ROS can play central roles in lipid peroxidation of endosomal membrane resulting in antigen escape to the cytosol and efficient antigen processing.^14^ Besides, ROS production could be a collateral effect in response to forced activation of the proteasomal machinery, which is often perceived as a danger “starvation” signal to the cell.^49^ As a result, affected cells trigger the activation of oxidative phosphorylation as a survival mechanism to support their metabolic needs through ATP production.^50^ Indeed, MitoSOX (mitochondria-specific) staining revealed an elevated ROS level in the MSC-UBvR-OVA group compared to the remaining MSC populations (Figures 4A and 4B) whereas no detectable signal was observed for cytoplasmic ROS in all groups using dihydroethidium (DHE) (Figures 4C and 4D). To assess the importance of ROS on antigen presentation, we next evaluated B3Z T cell activation following their co-culture with MSCs treated with various antioxidants. As shown in Figure 4E, treatment of both degron groups with MitoTEMPO (a blocker of mitochondrial ROS) decreased their activation ability, whereas no changes could be detected using N-acetylcysteine (NAC) (cysteine donor) or alpha-tocopherol (vitamin E derivative blocking lipid peroxidation). Based on the direct link between mitochondrial-derived ROS and antigen presentation, we next wondered whether ROS could impair antigen processing. We thus quantified OVA levels in the supernatant of gene engineered MSCs using an in-house ELISA. Interestingly, the supernatant of MSCs expressing the UBvR degron had lower OVA levels compared to control MSC-OVA, whereas their treatment with MitoTEMPO re-established OVA secretion to a level comparable to MSC-OVA (Figure 4F). These results highlight a direct link between ROS and degron-mediated degradation as they reveal their key role(s) in intracellular antigen processing.

### UBvR-engineered MSCs prime potent antitumoral responses

**Figure 5:**
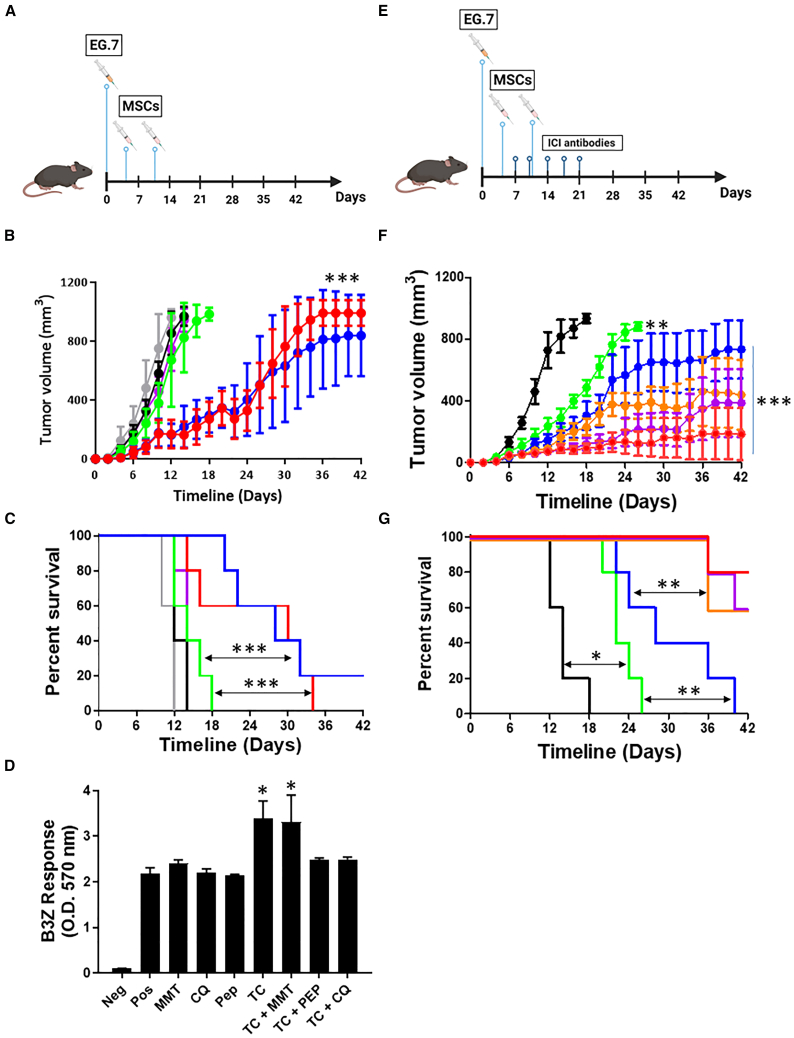
Therapeutic vaccination using the UBvR-engineered MSCs elicit potent antitumoral responses (A) Schematic diagram representing the vaccination approach used in the monotherapy study. (B) Tumor growth analysis overtime in response to MSC-OVA (green for female and purple for male) versus MSC-UBvR-OVA (blue for female mice and red for male mice). Animals receiving tumor cells only (control) are depicted by the black (female) and gray (male) lines. (C) Log rank survival of the experiment show in (B). (D) Antigen presentation assay using MSC-UBvR-OVA in response to multiple pharmacological treatments. (E) Schematic diagram representing the vaccination approach used in the combination study. (F) Tumor growth analysis overtime in response to anti-PD-1 (green), MSC-UbVR-OVA (blue), MSC-UBvR-OVA + anti-PD-1 (orange), TC-treated MSC-UBvR-OVA (purple), and TC-treated MSC-UBvR-OVA + anti-PD-1 (red). Animals receiving tumor cells only (control) as depicted by the black line. (G) Log rank survival of the experiment show in (F). For (B)–(D), (F), and (G), *n* = 5/group with ∗*p* < 0.05, ∗∗*p* < 0.01, and ∗∗∗*p* < 0.001. Data are represented as mean ± SD.

Given their impressive *in vitro* antigen presentation capacity, we next investigated the therapeutic potency of the MSC-UBvR-OVA population in animals with pre-established solid T cell lymphoma. Vaccine administration as a monotherapy (Figure 5A) controlled tumor growth (Figure 5B) resulting in a 20% survival 6 weeks post-vaccination with no major sex bias as both male and female mice responded in a similar fashion (Figure 5C). Since MSC-based therapies were previously reported to work in concert with various therapeutic modalities, we next compared the *in vitro* antigen-presenting capacity of MSC-UBvR-OVA following different pharmacological treatments including: (1) methylmalonic acid (MMA) (a booster of succinate build-up), (2) chloroquine (CQ) (to delay endosomal acidification in order to preserve antigens), (3) Pep (IWLTALKFLGKHAAKHEAKQQLSKL-C peptide, identified for its ability to break endosomal structures), (4) tranylcypromine (TC) to enhance H2-K^b^ cell surface stability, or (5) a combination of these treatments.^27^^,^^28^^,^^29^^,^^30^^,^^31^ As shown in Figure 5D, enhanced T cell activation was only observed when MSCs were treated with TC alone or in combination with MMA. Combining TC with Pep or CQ did not enhance T cell reactivity potentially due to antagonistic effects induced by mixing these agents (Figure 5D). Based on these observations, we next conducted a therapeutic vaccination trial using TC-treated MSCs in combination to anti-PD-1 (Figure 5E). Interestingly, the MSC-UBvR-OVA/anti-PD-1 combo group (orange) improves the antitumoral response compared to monotherapy (blue, Figure 5F) resulting in increased survival rates (60 versus 0%, respectively, Figure 5G). Notably, the use of TC-treated MSCs (purple) led to a response similar to the combo treatment group (orange) but was further improved when combined to anti-PD-1 (red, Figures 5F and 5G). These results suggest three important points. First, MSCs engineered to express a UBvR-degron fused to an antigen can trigger potent antitumoral responses. Second, pharmacological stimulation of these MSCs with TC can significantly enhance the therapeutic potency of the vaccine most likely due to enhanced cell surface H2-K^b^-peptide complexes as previously reported.^30^^,^^32^ Third, pharmacologically enhanced UBvR-expressing MSCs synergize with anti-PD-1 and elicit potent antitumoral responses.

### MSC-UBvR-OVA requires efferocytosis by endogenous phagocytes to elicit antigen-specific CD8 T cells

**Figure 6:**
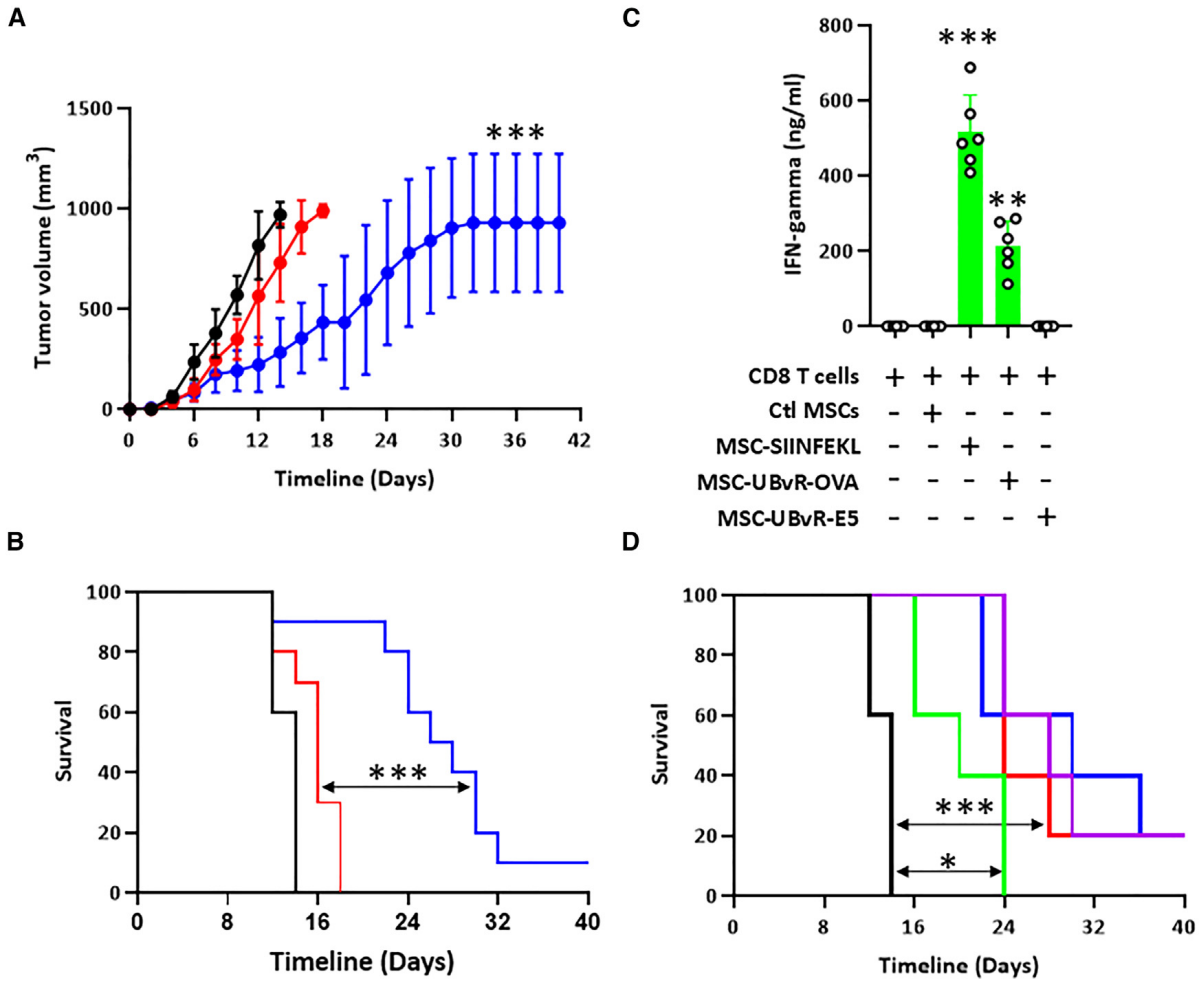
MSC-UBvR-OVA generates antigen-specific CD8 T cells via phagocyte efferocytosis (A) EG.7 tumor growth in mice vaccinated with MSC-UBvR-OVA (blue line) or with MSC-UBvR-E5 (encoding the HPV16 E5 as an irrelevant antigen). Control EG.7 is shown in black. (B) Log rank survival of the experiment show in (A). (C) An *in vitro* antigen presentation assay conducted using CD8 T cells isolated from MSC-UBvR-OVA-vaccinated mice and co-cultured with different MSC populations. (D) Log rank survival of mice undergoing phagocyte depletion using clodronate (green) or anti-CD11c depleting antibodies (red). Mice receiving control liposome are depicted by the purple line whereas mice receiving CD11c isotype antibody are represented by the blue line. Untreated control animals are represented by the black line. For (A) and (B) *n* = 10/group with ∗∗∗*p* < 0.001. For (C), *n* = 6/group with ∗∗*p* < 0.01 and ∗∗∗*p* < 0.001. For (D), *n* = 5/group with∗*p* < 0.05 and ∗∗∗*p* < 0.001. Data are represented as mean ± SD.

The previously observed *in vivo* potency suggests that destabilizing a given antigen using the UBvR degron can be indeed used as a modality to engineer cell-based vaccines capable of targeting cancer. However, it remains unknown if these engineered MSCs can directly behave as APCs when administered *in vivo* to trigger OVA-specific CD8 T cells. To first highlight the specificity of our cell vaccine, we conducted an additional vaccination study comparing MSC-UBvR-OVA to another cell product expressing the HPV16 E5 oncogene (MSC-UBvR-E5). Expression of a destabilized unrelated antigen such as HPV16 E5 (shown in red) did not trigger a meaningful anti-tumoral response (Figures 6A and 6B). To further re-enforce this concept, we conducted an *in vitro* antigen presentation assay using CD8 T cells isolated from MSC-UBvR-OVA-vaccinated mice (isolated 60 days post-second dosing). Not only did these CD8 T cells respond to SIINFEKL-pulsed MSCs (positive control) and MSC-UBvR-OVA (test condition), but no IFN-gamma production could be detected when these CD8 T cells were co-cultured with MSC-UBvR-E5 (Figure 6C). Now that the antigen specificity question is resolved, we next asked whether syngeneic MSC-UBvR-OVA could directly behave as APCs *in vivo* or if they rely on a third-party player to mediated T cell activation. Since past key studies highlighted that the *in vivo* mode of action of both syngeneic and allogeneic MSCs relies on efferocytosis mediated by endogenous phagocytes and/or involve DC cross-priming, we next tested our therapeutic vaccination approach in two different settings: (1) in animals undergoing phagocyte depletion using clodronate, or (2) following DC depletion using anti-CD11c antibodies.^29^^,^^51^^,^^52^ Interestingly, depletion of DCs had no impact on animal survival compared to MSC-UBvR-OVA-vaccinated animals injected with isotype antibodies (red versus blue line) indicating that DCs are not cross-primed *in vivo* following MSC-UBvR-OVA administration (Figure 6D). On the other hand, clodronate administration prior to MSC-UBvR-OVA injection greatly impairs the vaccine potency as all animals succumb by day 24 (green line, Figure 6D). The sum of these results indicate that MSC-UBvR-OVA vaccination can indeed elicit an OVA-specific CD8 T cell response but requires phagocyte-mediated efferocytosis to mediate such a response.

## Discussion

Several studies indicated that, under steady state, the immunopeptidome (the collective pool of peptides presented by MHC I) derives from the rapid degradation of newly synthesized polypeptides containing normal short-lived proteins as well as defective ribosomal products.^17^^,^^18^^,^^19^^,^^20^^,^^21^ For this process to occur, however, ubiquitin molecules need to be covalently attached to “client” proteins by enzymes of the ubiquitin-proteasome system.^2^^,^^3^^,^^8^^,^^9^^,^^10^ Although ubiquitination can target any protein and exhibit various functions, degron motifs act as “magnets” to ubiquitin molecules resulting in enhanced proteasomal degradation.^10^^,^^11^^,^^12^^,^^13^ We thus evaluated a versatile, easy-to-use approach where an intracellular transgene is destabilized using two degron sequences. Compared to the ubiquitin-independent degron RNP4 1–80, OVA degradation was more efficient when fused to the ubiquitin-dependent UBvR degron repeats. The net outcome culminates in forced degradation of the transgene product consequently eliciting powerful CD8 T cell activation capable of controlling tumor growth in animals with pre-established lymphoma.

The most interesting observation made from this study was the improved capacity of MSC-UBvR-OVA to enhance the activation of CD8 T cells without fluctuating the expression of genes related to antigen processing and loading or ER stress. This enhanced antigen presentation was also independent of increased cell surface expression of MHCI. Based on these observations, an immunopeptidome study was conducted to mine the peptide repertoire of these engineered cells. Interestingly, an increase in the relative intensity of the SIINFEKL peptide and the appearance of an additional 10-mers OVA-derived peptide were both identified in the UBvR group, which implies two things. First, the ubiquitin-independent degron is less active at degrading proteins, at least in MSCs, while the UBvR degron specifically enriched the presence of the SIINFEKL peptide. This begs the question: is OVA secretion affected by the inclusion of the UBvR degron? When assessed by ELISA, the OVA levels in the supernatant of the MSC-OVA group had almost three times more OVA than the MSC-UBvR-OVA group. Interestingly however, OVA destabilization by UBvR degron relied on the production of ROS as their neutralization impaired antigen presentation while increasing OVA secretion to a level comparable to the control MSC-OVA group. The role played by ROS in this context is 2-fold. First, ROS production was detected with both tested degrons suggesting a possible link between enhanced proteasomal function and mitochondrial activity. In fact, the proteasomal machinery requires excessive energy in the form of ATP during polypeptide entry, deubiquitinating and processing. Sustained proteasomal stimulation could therefore be perceived as a “starvation” signal due to exacerbated proteolysis activities triggering the mitochondria to activate oxidative phosphorylation.^48^ However, this does not explain the neutralizing effect of ROS on antigen processing and secretion. As such, it is possible to stipulate that ROS production contributes to causing protein damage further enhancing ubiquitination of OVA and potentially other intracellular proteins targeting them to proteasomal degradation. Further studies are therefore warranted to better decipher the exact effect of ROS in the degradation of destabilized degron-OVA and/or other target proteins.

The herein study relies on two distinct technologies: forced intracellular degradation of expressed xenoantigens and the use of MSC as a vaccination platform. As MSC-based vaccination strategies were previously reported, this modality is an “add-on” to our current pipeline. These data further confirm that MSC can be engineered to express therapeutically relevant antigens to target pre-established tumors. In addition, our model conveys a strategy where MSCs expressing a destabilized form of a given antigen could be used to identify immunogenic peptide sequences selectively presented on their cell surface MHCI to activate CD8 T cells. Such approach can lead to the establishment of a vaccine discovery program possibly amenable to cancer and emerging pandemics.

### Limitations of the study

Despite the impressive therapeutic potency observed using the degron strategy to trigger antigen degradation, this approach is mainly limited by the targeted antigen of choice. For instance, potent anticancer effects can be achieved only if a tumor-specific antigen or a newly identified neoantigen are used. This means that the degron technology is suitable as a personalized vaccination approach as patient-specific antigens must be used. This limitation, on the other hand, does not apply to antigens derived from infectious diseases as they are considered as non-self-antigens.

## Resource availability

### Lead contact

Further information and requests for resources and reagents should be directed to and will be fulfilled by the lead contact, Moutih Rafei (moutih.rafei.1@umontreal.ca).

### Materials availability

All unique/stable reagents generated in this study are available from the lead contact with a completed materials transfer agreement and may require a payment a if there is potential for commercial application.

### Data and code availability

• The mass spectrometry proteomics data have been deposited at the PRIDE partner repository as [Forced Intracellular degradation of Xenoantigens as a modality for cell-based cancer immunotherapy: PXD059791] and are publicly available as of the date of publication.
• This paper does not report original code.
• Any additional information required to reanalyze the data reported in this paper is available from the lead contact upon request.

## Acknowledgments

We wish to thank all technicians from the IRIC animal facility for their kind support regarding some of the *in vivo* experiments. Proteomics analyses were performed by the Center for Advanced Proteomics Analyses, a Node of the Canadian Genomic Innovation Network that is supported by the Canadian Government through Genome Canada. Some of the figures displayed in the manuscript were generated using the Biorender drawing tools. RF is the recipient of a Cole Foundation Doctoral Research Award. G.A.M. is the recipient of a Natural Sciences and Engineering Research Council post-doctoral fellowship award (PDF-587335-2024). This work was supported by a Canadian Institutes of Health Research project grant (PJT-186233) and by an operating grant (#834469) from the 10.13039/100009326Cancer Research Society.

## Author contributions

J.P.B. and R.F. designed and conducted most of the *in vitro* assays, *in vivo* experiments and analyzed data. J.A., G.A.M., M.A.E., P.M., and M.L. designed and executed some *in vitro* experiments. M.P.G. and S.T. contributed to data analysis and generation of schematic diagrams/cartoons. N.E.-H. conducted the immunopeptidome analysis. M.R. conceived and supervised the project, analyzed all collected data, and wrote the first draft of the manuscript. All authors contributed to manuscript editing.

## Declaration of interests

All authors declare no competing financial interest.

## STAR★Methods

### Key resources table

**Table:** 

REAGENT or RESOURCE	SOURCE	IDENTIFIER
**Antibodies**		

Alexa Fluor® 647 Mouse Anti-Mouse H-2K[b]	BD Biosciences	Cat# 562832, RRID:AB_2737824
Recombinant MAb anti-mouse PD-1 (CD279)	BioXCell	Cat# CP157, RRID:AB_2927526
Anti-CD11c (Clone RMP1-14)	BioXCell	Cat#: BE0146, RRID:AB_10949053
Goat Anti-Mouse IgG HRP	R&D systems	Cat#: HAF007; RRID:AB_357234
Monoclonal Anti-chicken egg Albumin	Sigma-Aldrich	Cat#: A6075-2ML, RRID:AB_258279

**Chemicals, peptides, and recombinant proteins**		

Accutase®	Sigma-Aldrich	Cat#: A6964
Chlorophenol red-β-D-galactopyranoside (CPRG)	Sigma-Aldrich	Cat#: 10884308001
Chloroquine (CQ)	InvivoGen	Cat# tlrl-chq-4
Dihydroethidium (DHE)	Invitrogen	Cat#: D11347
Diphenyleneiodonium Chloride (DPI)	Sigma-Aldrich	Cat#: D2926-10MG
DL-alpha-Tocopherol	Sigma-Aldrich	Cat#: PHR1031-500MG
MitoTEMPO	Sigma-Aldrich	Cat# SML0737-5MG
MitoSOX	Invitrogen	Cat#: M36008
N-Acetyl-L-cysteine (NAC)	Sigma-Aldrich	Cat#: A7250
Tranylcypromine (TC)	Invitrogen	Cat#: 616431-500MG
Methylmalonic acid (MMA)	ThermoFisher	Cat#: 127090050
SIINFEKL peptide	GenScript	Cat#: RP10611
IWLTALKFLGKHAAKHEAKQQLSKL-C peptide (Pep)	GenScript	N/A - synthesized
3,3′,5,5′-Tetramethylbenzidine (TMB)	Sigma Aldrich	Cat#T0440

**Critical commercial assays**		

RNeasy Mini Kit	QIAGEN	Cat# 74104
EasySep Mouse CD8a Positive Selection Kit II	StemCell Technologies	Cat#:18953
Mouse IFN-gamma Quantikine ELISA Kit	R&D systems	Cat#: MIF00-1
RNeasy Mini Kit	QIAGEN	Cat# 74104
EasySep Mouse CD8a Positive Selection Kit II	StemCell Technologies	Cat#:18953

**Deposited data**		

The mass spectrometry proteomics data	ProteomeXchange Consortium via the PRIDE partner repository	Project accession#: PXD059791

**Experimental models: Cell lines**		

Mouse: E.G7-OVA [derivative of EL4]	ATCC	Cat# CRL-2113; RRID: CVCL3505
Mouse: B3Z	Gift from Dr. Michel Desjardins	N/A

**Experimental models: Organisms/strains**		

Mouse: C57BL/6NCrl	Charles River	Strain code: 027
Mouse: OT1 (C57BL/6-Tg(TcraTcrb)1100Mjb/J)	The Jackson Laboratory	Strain code: 003831

**Software and algorithms**		

FlowJo™ v10	FlowJo	https://www.flowjo.com/solutions/flowjo/downloads
GraphPad Prism	Dotmatics	https://www.graphpad.com/
BioRender	BioRender	https://www.biorender.com/
Bioinformatics software(s)	R statistical programming, Other packages: Ggplot2 ClusterProfiler	https://cran.r-project.org/web/packages/ggplot2/index.html https://bioconductor.org/packages/release/bioc/html/clusterProfiler.html

**Other**		

Amicon® Ultra-15 Centrifugal Filter Unit	Millipore	Cat# UFC910024
96-well microtiter Nunc MacIvor plates	Thermo Fisher Scientific	Cat#: 442404

### Experimental model and study participant details

#### Animals and ethics

For all experiments, six- to eight-week-old male and female C57BL/6 were purchased from Charles River (Senneville, QC, Canada). Female OT-1 mice were purchased from the Jackson Laboratory (Bar Harbor, ME, USA). The mice were housed and maintained in accordance with the guidelines approved by the Animal Care Committee of Université de Montréal in a pathogen-free environment at the animal facility of the Institute for Research in Immunology and Cancer (IRIC). All experimental procedures were approved by the Animal Committee of Université de Montréal and were carried out in accordance with relevant guidelines and regulations (22-065).

#### Generation and validation of murine bone-marrow (BM)-derived MCSs

Bone marrow derived MSCs were obtained as previously detailed.^27^ Briefly, the bone marrow of 6-8 weeks old female C57BL/6 mice was flushed from the femur into 10 cm^2^ cell culture dish with Alpha Modification of Eagle’s Medium (AMEM) supplemented with 10% FBS, and 50 U/mL Penicillin-Streptomycin. Non-adherent cells were removed after 24 hours followed by gently changing the media every 3 to 4 days. The cells were then detached and collected using 0.25% Trypsin and expanded until reaching 80% confluency. Once a homogeneous population was obtained and confirmed for the expression of MSC surface markers by flow cytometry, the cells were expanded and stored in liquid nitrogen at passage number 9 – 10.^53^ The differentiation capacity of generated MSCs was evaluated by inducing their differentiation into osteocytes and adipocytes as reported previously.^53^ To stimulate osteogenic differentiation, the MSCs were seeded at 60% confluency followed by 3-4 weeks culture in AMEM media supplemented with 10% FBS, β-glycerol phosphate (10 mM), dexamethasone (10^-8^ M), and ascorbic acid 2-phosphate (5 μg/mL). The media was changed two to three times a week. Confirmation of osteogenic differentiation was done by staining the calcium deposits using Alizarin Red S. After removing culture media and washing with phosphate-buffered saline (PBS), the cells were incubated for 5 minutes in 2% Alizarin Red S solution (pH adjusted to 4.1 using ammonium hydroxide), then rinsed with distilled H_2_O. To trigger MSCs transformation into adipocytes, the cells were seeded at 50% confluency and cultured in AMEM supplemented with 10% FBS, indomethacin (46 μM), 3-isobutyl-methylxanthine (0.5 mM), dexamethasone (1 μM), and insulin (10 μg/mL) for 7 days, during which the media was changed twice. Confirmation of adipocytes formation was done at the end of 7 days by staining oil droplets within the cells using Oil Red O. After removing culture media, the cells were fixed for 1 hour at room temperature using 4% paraformaldehyde. The cells were then stained for 10 minutes using Oil Red O solution (3.75% Oil Red O. in isopropanol mixed with 2 parts distilled H_2_O). At the end of staining, the cells were rinsed using distilled H_2_O^53^ and visualized via transmitted light microscope and imaged using EVOS® FL cell imaging microscope (Thermo Fisher Scientific).

#### Engineering of primary murine BM-derived MSCs

To engineer MSCs to express gene of interest a construct was designed containing the cDNA of the gene of interest with or without being preceded by the tested degron sequence. The designed construct was synthesized, sub-cloned into the AP2 retroviral plasmid and sequenced By Genscript. To generate the virus containing the gene insert, the sub-cloned AP2 was co-transfected with VSV-G encoding viral coating protein into the GP2-293 packaging cell line using PolyFect® following the manufacturer's instructions. The culture media supernatants containing the virus collected 48 hours post-transfection was centrifuged at 1500 rpm for 5 min at 4°C to remove cell debris. The collected viral preparations were then added with a multiplicity of infection of 2 onto MSCs previously plated at a 50-60% confluency. The AP2 construct contains the enhanced green fluorescence protein (eGFP) as a marker for gene expression,^54^ accordingly, transduction efficiency was confirmed through assessment of GFP expression by flow-cytometry.

#### Cell lines

E.G7 tumor cells (generated via EL4 transfection to express the OVA protein – originating from a syngeneic C57BL/6 mouse) were purchased from ATCC. B3Z cells (T-cell hybridoma expressing H2-K^b^/SIINFEKL on their surface- originating from an allogenic Balb/c mouse) were a generous gift from Dr. Michel Desjardins (Université de Montréal, Montreal, QC, Canada). Both cell lines were maintained as previously described.^55^ Briefly, E.G7 and B3Z cells were cultured in RPMI 1460 supplemented with 10% FBS, 50 U/mL Penicillin-Streptomycin, 2 mM L-glutamine, 10mM HEPES, 1mM Sodium Pyruvate, and 0.5 mM β-Mercaptoethanol. E.G7 cells were kept under selection using 0.4 mg/ml of G418. All cells were maintained at 37°C in a 5% CO2 incubator. All cell culture media and reagents were purchased from Wisent Bioproducts (St-Bruno, QC, Canada).

#### Therapeutic vaccination studies

For therapeutic vaccination, male (*n*=5/group) or female C57BL/6 mice (*n=*5/group) received a subcutaneous (SC) injection of 5 × 10^5^ E.G7 cells at day 0. Three days later (appearance of palpable tumors ∼ 35-50 mm^3^), mice were subcutaneously (SC)-injected with a 100 μl of sterile PBS containing 5 × 10^5^ MSC-OVA, MSC-UBvR-OVA, or TC-treated MSC-UBvR-OVA (two injections one week apart) on the counter lateral flank (distal sites to avoid direct interference with tumor growth in an antigen-independent manner). Control animals received 5 × 10^5^ tumor cells alone. Treated animals were followed thereafter for tumor growth using a digital caliper. For therapeutic vaccination in combination with the anti-PD-1 immune-checkpoint inhibitor, mice received intraperitoneal (IP) injections of the antibody or its isotype control at 200 μg/per dose every 2 days for a total of 6 doses over two weeks. To block efferocytosis, C57BL/6 mice (*n*=10/group) with pre-established E.G7 tumors were IP-injected with liposome-clodronate or control liposome (50 μl/20 g mouse) one day prior to each vaccination (two given in total).^29^ For blocking DC cross-priming, animals were IP-injected with 30 μg of anti-CD11c antibodies or isotype control every 2 days for a total of three doses over 1 week prior to E.G7 transplantation and MSC-UBvR-OVA vaccination. The same process was repeated the following week for anti-CD11c administrations prior to second dosing.

### Method details

#### Antigen presentation assay

To assess the antigen presentation ability of gene-engineered MSCs, MSCs were seeded in a 24-well plate at 25 × 10^3^ cells per well. On the following day, the cells were washed with PBS, then 5 × 10^5^ B3Z cells were added to each well. Control MSCs pulsed for 3 hours with the SIINFEKL peptide (0.1 μg/ml) were used as a positive control for the assay. The co-culture was incubated for 17-19 hours before the media was removed, cells washed once with PBS then lysed using lysis buffer (tris base, CDTA, glycerol and triton X-100) and shaken for 20 minutes at room temperature. Cell lysate is then incubated with a CPRG solution (containing CPRG, disodium phosphate, monosodium phosphate, potassium chloride, magnesium sulfate) protected from light- for 24 hours at 37°C. The optical density signal was detected at wavelength 570 nm using a SynergyH1 microplate reader (Biotek, Winooski, VT, United States). For antigen presentation assays in the presence of pharmacological treatments, MSCs were either treated with TC (500 μM), CQ (10 μM), MMA (5 μM) or Pep (100 μM) 12 hours prior to assessing their ability to activate T cells as detailed above.

For the assay using vaccinated- or OT-1-derived CD8 T cell, engineered MSCs were seeded at a density of 25 × 10^3^ cells per well in a 24-well plate. The cells were washed on the following day prior to adding 10^6^ cells/ml of purified OT-1 CD8 T cells. The cells were obtained from the spleen of OT-1 mouse using the CD8α^+^ positive isolation kit according to the manufacturer’s protocol. For positive control, non-engineered MSCs were pulsed with 0.1 μg/ml of the SIINFEKL peptide for 24 hours prior to adding OT-1 CD8 T cells. After 72 hours, supernatants were collected, and centrifuged for 5 min at 1500 rpm, 4°C. Clear supernatants were used to quantify IFN-gamma levels by ELISA according to manufacturer instruction (R&D Systems).

#### Immunopeptidome studies

Assessment of peptide repertoire presented on the surface of MSCs was conducted as previously described.^27^ Briefly, MSCs were detached using Accutase®, the cell pellets were washed 3 times with PBS prior to snap-freeze in liquid nitrogen. Fifty million cells were pelleted and flash frozen prior to lysing using a 1% CHAPS-based buffer and cleared by centrifugation. Clarified lysates were incubated with 200 μg Pan-H2 and H2-Kb linked to CNBr-activated sepharose overnight to immunoprecipitate mouse MHC class I, then washed with lysis buffer followed by Tris-HCl with decreasing NaCl concentrations. The final elution was carried out in LoBind Eppendorf tubes using 1% TFA. Peptides were concentrated and desalted using solid-phase extraction (SPE) with an Empore C18 plate. Peptides were loaded directly and eluted using 80/20 acetonitrile/water (0.1% TFA). Eluted peptides were lyophilized and reconstituted in 0.1% TFA. Peptides (50% per sample) were analyzed by nano LC/MS/MS using a Thermo Vanquish Neo system interfaced to a ThermoFisher Ascend mass spectrometer. Peptides were loaded on a trapping column and eluted over a 75 μm analytical column at 300 nL/min; both columns were packed with Jupiter C18 resin (Phenomenex). A 2-hour gradient was employed. The mass spectrometer was operated using a custom data-dependent method, with MS performed in the Orbitrap at 120,000 FWHM resolution and sequential MS/MS performed using high resolution HCD in the Orbitrap at 30,000 FWHM resolution. All MS data were acquired from m/z 350-760 (Class I). A 3s cycle time was employed for all steps. Peptide analysis was conducted using the free online analysis tools GibbsCluster and NetMHCpan to stratify the peptides identified in the immunopeptidome sequencing. Binding affinity predictions are classified by the percentage rank with strong binding (SB) = <0.5%; moderate biding (MB) = 0.5% - 2.0%; and weak binding (NB) = >2.0%.

#### Quantification of OVA secretion by ELISA

To quantify OVA in the supernatant of MSCs expressing OVA or UBvR-OVA in the absence or presence of MitoTEMPO, 10^6^ MSCs were cultured in serum-free media. After 24 hours, the supernatant was collected and concentrated 30 folds using a centurion prior to protein quantification using an in-house developed ELISA. Briefly, a 96-well microtiter Nunc MacIvor plates (Thermo Fisher Scientific; Cat#: 442404) was coated with 100 μL of the concentrated cell supernatant and left coating overnight at 4°C. After washing the following day, the plate was blocked using a 150 μL solution containing 3% milk powder diluted in PBS and left for 1 hour at room temperature. After a series of washes, the primary monoclonal anti-OVA (1:500) (Sigma-Aldrich, A6075) was incubated for 2 hours at room temperature. Following that step and subsequent washes, 100 μL of the secondary anti-mouse IgG HRP conjugated antibody (R&D Systems HAF007) (1:1000) was added for another 2 hours at room temperature. The reaction was revealed, after washing three times, by adding 100 μL of 3,3′,5,5′-Tetramethylbenzidine (TMB) (Sigma Aldrich, T0440) left for 30 min at room temperature. The reaction was stopped by adding 50 μl of 1M H_2_SO_4_ to each well and the absorption measured using a Synergy H1 plate reader from Bio Tek set to 450 nm and 570 nm wavelength. The results were presented as OVA quantity per 10^6^ cells per 24 hours.

#### Evaluating ROS production and their effect on antigen presentation

Production of ROS was evaluated by flow-cytometry using both MitoSOX™ and dihydroethidium (DHE). Briefly, 2 × 10^4^ MSCs were collected, washed, and stained at 37°C for 30 minutes using MitoSOX™ according to manufacturer instructions or 10 μM DHE. After the staining period was completed, the cells were washed and analyzed by flow-cytometry within 1 hour. To evaluate the effect of ROS neutralization on antigen presentation, the same approach described above was performed except that the cells were treated with NAC (5 mM) as a general ROS inhibitor, MitoTEMPO (10 μM) as a specific mitochondrial ROS inhibitor, or α-tocopherol (2000 μM) as a blocker for lipid peroxidation. Treated cells were then evaluated by antigen presentation assay as detailed above to assess any changes in OVA production, processing or ability to present the SIINFEKL peptide to B3Z cells.

### Quantification and statistical analysis

#### RNA extraction and bioinformatic analysis

Total RNA was isolated from 10^6^ cells of each MSC population using the RNeasy® mini kit (QIAGEN) according to manufacturer’s instructions. Quality of the extracted RNA was assessed by nanodrop using 260/280 and 260/230 ratios. Quantification of total RNA was made by QuBit (ABI) and 50 ng of total RNA was used for qPCR at the Institute for Research in Immunology and Cancer’s Genomics Platform (IRIC). Transcript levels were then presented in the form of a heatmap generated using the GraphPad Prism 10 software (version 10.2.1).

#### Peptides bioinformatic analysis

All computational analyses and plotting were done in the R programming language. Reactome pathways were downloaded from MsiGDB,^56^ and corresponding human genes were converted to mouse orthologs. Enrichment analysis was done with the clusterProfiler package,^57^ and custom R scripts. Intersection plots were made with the UpSetR package using default parameters (Conway, Lex & Gehlenborg 2017).

#### Statistical analysis

*p-*values were calculated using one-way analysis of variance (ANOVA) or Log-rank test for animal survival experiments. Results are represented as average mean with standard deviation (S.D.) error bars, and statistical significance is represented with asterisks: ∗*p* ˂ 0.05, ∗∗*p* ˂ 0.01, ∗∗∗*p* ˂ 0.001.
