# Supplementary material for: Forced intracellular degradation of xenoantigens as a modality for cell-based cancer immunotherapy
Source: iScience. 2025 Feb 4;28(3):111957. doi: 10.1016/j.isci.2025.111957 (PMC11889607; doi:10.1016/j.isci.2025.111957)
Supplement: Document S1. Figures S1–S3 [file mmc1.pdf]

iScience, Volume *28*

Supplemental information

Forced intracellular degradation of xenoantigens as a modality for cell-based cancer immunotherapy

Jean Pierre Bikorimana, Roudy Farah, Jamilah Abusarah, Gabrielle Arona Mandl, Mohamed Ali Erregragui, Marina Pereira Gonçalves, Sebastien Talbot, Perla Matar, Malak Lahrichi, Nehme El-Hachem, and Moutih Rafei

A) OVA cDNA

MGSIGAASMEFCFDVFKELKVHHANENIFYCPIAIMSALAMVYLGAKDSTRTQINKVVR
FDKLPGFGDSIEAQCGTSVNVHSSLRDILNQITKPNDVYSFSLASRLYAEERYPILPEYLQC
VKELYRGGLEPINFQTAADQARELINSWVESQTNGIIRNVLQPSSVDSQTAMVLVNAIVF
KGLWEKTFKDEDTQAMPFRVTEQESKPVQMMYQIGLFRVASMASEKMKILELPFASGT
MSMLVLLPDEVSGLEQLESIINFEKLTEWTSSNVMEERKIKVYLPRMKMEEKYNLTSVL
MAMGITDVFSSSANLSGISSAESLKISQAVHAAHAEINEAGREVVGSAEAGVDAASVSEE
FRADHPFLFCIKHIATNAVLFFGRCVSP

B) 3xUBvR-OVA cDNA

MTSQIFVKTLTGKTITLEVEPSDTIENVKAKIQDKEGIPPDQQRLIFAGKQLEDGRTLSDY
NIQKESTLHLVLRLRGVRASASQIFVKTLTGKTITLEVEPSDTIENVKAKIQDKEGIPPDQQ
RLIFAGKQLEDGRTLSDYNIQKESTLHLVLRLRGVRASASQIFVKTLTGKTITLEVEPSDTI
ENVKAKIQDKEGIPPDQQRLIFAGKQLEDGRTLSDYNIQKESTLHLVLRLRGVRASASSM
GSIGAASMEFCFDVFKELKVHHANENIFYCPIAIMSALAMVYLGAKDSTRTQINKVVRF
DKLPGFGDSIEAQCGTSVNVHSSLRDILNQITKPNDVYSFSLASRLYAEERYPILPEYLQC
VKELYRGGLEPINFQTAADQARELINSWVESQTNGIIRNVLQPSSVDSQTAMVLVNAIVF
KGLWEKTFKDEDTQAMPFRVTEQESKPVQMMYQIGLFRVASMASEKMKILELPFASGT
MSMLVLLPDEVSGLEQLESIINFEKLTEWTSSNVMEERKIKVYLPRMKMEEKYNLTSVL
MAMGITDVFSSSANLSGISSAESLKISQAVHAAHAEINEAGREVVGSAEAGVDAASVSEE
FRADHPFLFCIKHIATNAVLFFGRCVSP

C) RPN4 1-80-linker-OVA cDNA

MASTELSLKRTLTDILEDELYHTNPGHSQFTSHYQNYHPNASITPYKLVNKNKENNTFTW
NHSLQHQNESSAASIPPQQTASASSMGSIGAASMEFCFDVFKELKVHHANENIFYCPIAIM
SALAMVYLGAKDSTRTQINKVVRFDKLPGFGDSIEAQCGTSVNVHSSLRDILNQITKPND
VYSFSLASRLYAEERYPILPEYLQCVKELYRGGLEPINFQTAADQARELINSWVESQTNGII
RNVLQPSSVDSQTAMVLVNAIVFKGLWEKTFKDEDTQAMPFRVTEQESKPVQMMYQIG
LFRVASMASEKMKILELPFASGTMSMLVLLPDEVSGLEQLESIINFEKLTEWTSSNVMEER
KIKVYLPRMKMEEKYNLTSVLMAMGITDVFSSSANLSGISSAESLKISQAVHAAHAEINE
AGREVVGSAEAGVDAASVSEEFRADHPFLFCIKHIATNAVLFFGRCVSP

D) 3xUBvR-E5 cDNA

MTSQIFVKTLTGKTITLEVEPSDTIENVKAKIQDKEGIPPDQQRLIFAGKQLEDGRTLSDY
NIQKESTLHLVLRLRGVRASASQIFVKTLTGKTITLEVEPSDTIENVKAKIQDKEGIPPDQQ
RLIFAGKQLEDGRTLSDYNIQKESTLHLVLRLRGVRASASQIFVKTLTGKTITLEVEPSDTI
ENVKAKIQDKEGIPPDQQRLIFAGKQLEDGRTLSDYNIQKESTLHLVLRLRGVRASASSM
TNLDTASTTLLACFLLCFCVLLCVCLLIRPLLLSVSTYTSLIILVLLLWITAASAFRCFIVYII
FVYIPLFLIHTHARFLIT*

Figure S1. Amino Acid sequences for the three OVA and E5 cassettes used in this study, Related to Figure 1. [Characterizing the antigen presenting properties of gene-engineered MSCs]. A) Sequence of non-modified OVA. **B)** Sequence of UBvR-OVA. **C**) Sequence of RNP-OVA. **D)** Sequence of UBvR-E5.

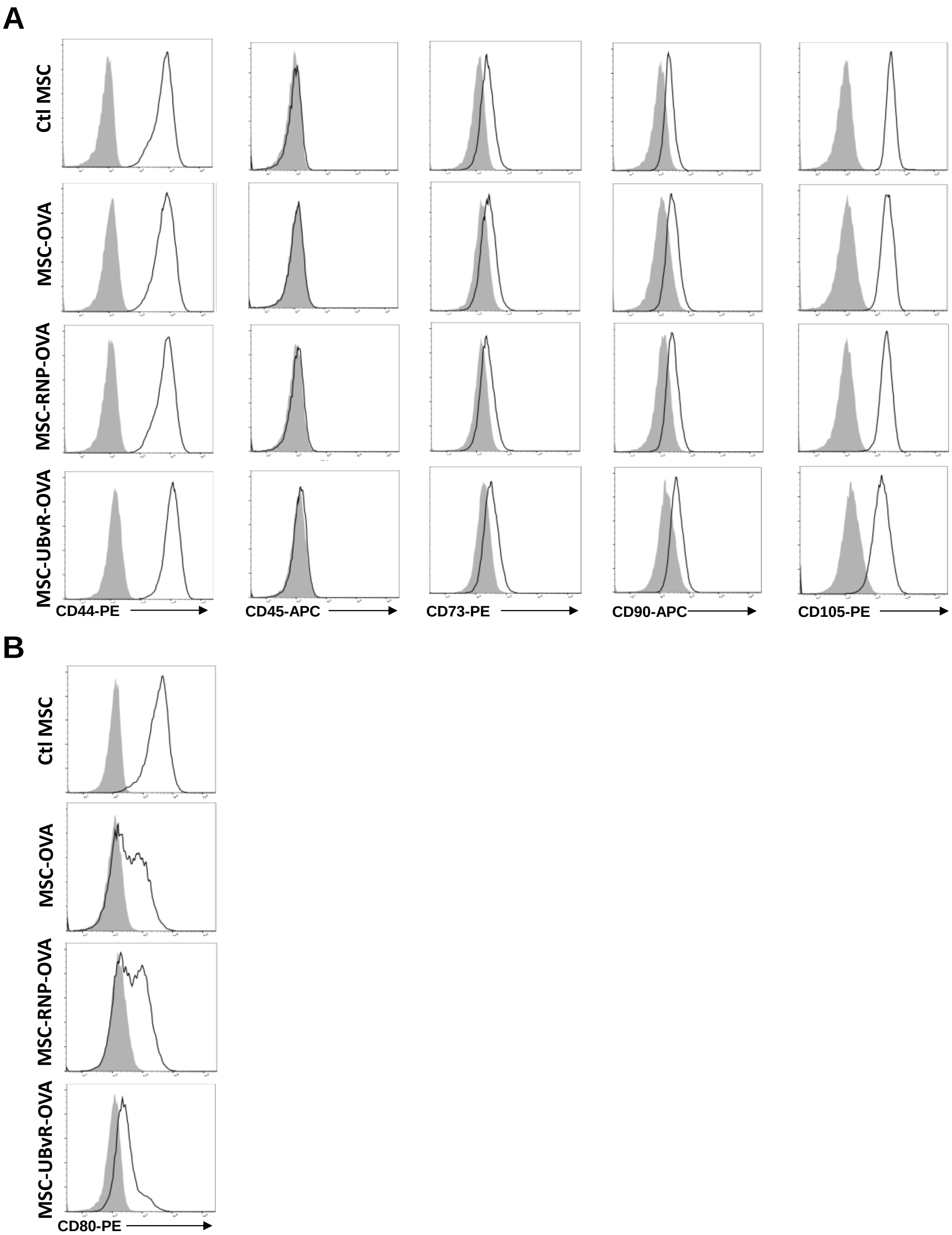

Figure S2. Phenotypic analysis of engineered MSCs, Related to Figure1. [Characterizing the antigen presenting properties of gene-engineered MSCs]. A) Analysis of the innate markers normally used to confirm the identity of MSCs. Filled grey histograms represent isotype controls. **B)** Same as (A) except done on the co-stimulatory molecule CD80.

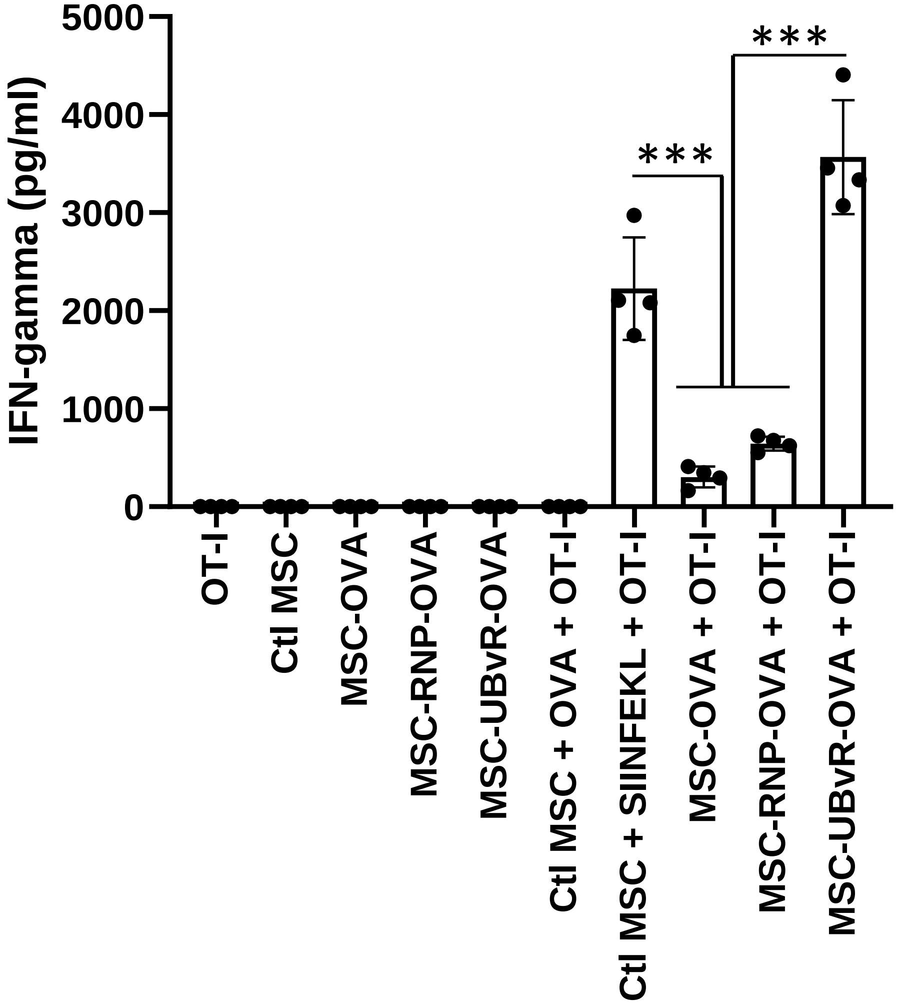

Figure S3. Antigen presentation assay using OT-I-derived primary CD8 T cells, Related to Figure1. [Characterizing the antigen presenting properties of gene-engineered MSCs]. Consistent with the B3Z results, OT-I co-cultured with MSC-UBvR-OVA lead to the highest IFN-gamma production. The different conditions are as follow: i) Ctl MSC: MSCs that are not transduced or pulse, ii) MSC-OVA: MSCs that are retrovirally transduced to express the OVA protein, iii) MSC-RNP-OVA: MSCs that are retrovirally transduced to express the RNP-OVA sequence, iv) MSC-UBvR-OVA: MSCs that are retrovirally transduced to express the UBvR-OVA sequence, v) Ctl MSC + OVA: MSCs pulsed with the OVA protein, and iv) Ctl MSC + SIINFEKL: MSCs pulsed with the SIINFEKL peptide. For this assay, n=4/group with ***P<0.001.
